# A complex case of necrobiotic xanthogranuloma with IgG‐kappa paraproteinemia: Disease regression achieved with melphalan and prednisone combination therapy

**DOI:** 10.1002/ccr3.6554

**Published:** 2022-11-15

**Authors:** Jacques A. J. Malherbe, Julian Cooney

**Affiliations:** ^1^ Department of Haematology Fiona Stanley Hospital Murdoch Western Australia Australia; ^2^ School of Biomedical Sciences University of Western Australia Crawley Western Australia Australia

**Keywords:** melphalan, necrobiotic xanthogranuloma, paraproteinemia, prednisone

## Abstract

Necrobiotic xanthogranuloma (NXG) is a rare dermatosis that is often associated with monoclonal paraproteinemia and hematological malignancies. We report the rare case of an 84‐year‐old gentleman with extensive truncal NXG and IgG‐kappa paraproteinemia who achieved significant disease regression following six cycles of combination melphalan/prednisone therapy.

## Graphical abstract

Skin infiltration by necrobioc xanthogranuloma: Before treatment above in A, after treatment in B, with paraprotein response to therapy.

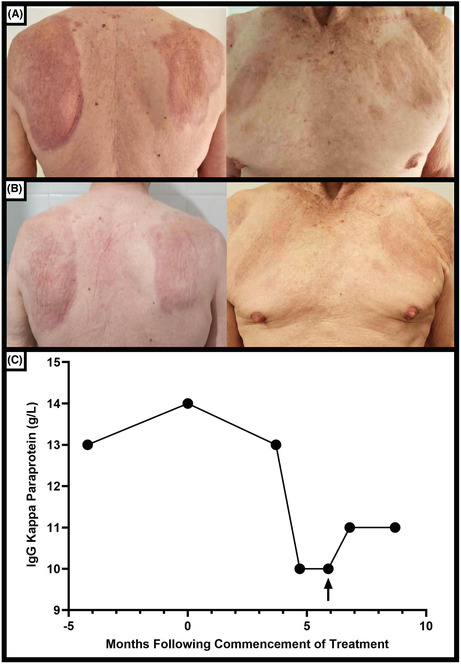

## 1 INTRODUCTION

Necrobiotic xanthogranuloma (NXG) is a rare, non‐Langerhans inflammatory histiocytosis that involves the dermal and subdermal layers of the skin.^1^, ^2^ It has a predilection for periorbital locations but can form coalescing papules and indurated plaques across the entire trunk and limbs. Although initial lesions are often painless, progression to large plaques may be associated with severe ulceration, telangiectasia, pruritus and/or skin atrophy, predisposing patients to skin breakdown, pain, and infection.^1^, ^2^ The disease is frequently associated with a monoclonal paraprotein in up to 80% of patients, of which IgG‐kappa paraproteinemia is the most commonly observed.^1^, ^2^ Progression to multiple myeloma occurs in up to 10% of patients, with the remainder at risk for other hematological malignancies, e.g., non‐Hodgkin's lymphoma, amyloidosis, and myelodysplasia.^1^ Some patients may also show visceral involvement. Biochemically, NXG may also present with hyperlipidemia, cryoglobulinaemia, leukopenia, and hypocomplementemia.^1^, ^2^

Treatment recommendations detailing the duration, dose, and drugs required, in addition to the clinical monitoring criteria to determine disease recurrence in NXG patients are limited due to the inherent paucity of clinical publications regarding the disease. Several case reports and small, retrospective case series have shown variable clinical responses to topical steroid applications, interferon‐α, corticosteroid therapy (either alone or in combination with alkylating agents, e.g., chlorambucil, cyclophosphamide, melphalan), infliximab, intravenous immunoglobulin, radiotherapy, surgery, and extracorporeal photopheresis.^1^, ^2^, ^3^, ^4^, ^5^, ^6^, ^7^, ^8^, ^9^, ^10^, ^11^, ^12^, ^13^ Two different treatment guidelines have been proposed.^1^, ^10^ Migel et al.^1^ consider the coexistence of a hematological malignancy to direct treatment. For exclusive dermatological disease, surgery is recommended as the first line approach, whereas for those with a concurrent haematologic neoplasm, chlorambucil monotherapy or in combination with prednisone is suggested. Treatments for further relapses of the latter are stratified according to the localisation of NXG lesions to the face/periorbital regions or elsewhere along the body. Although more recent treatment guidelines from Hilal et al.^10^ also consider the presence of a hematological malignancy as an important factor, treatment options are less prescriptive. These guidelines also consider the extent, localisation and clinical characteristics of the NXG lesions to guide therapy. However, neither treatment guideline provides details regarding the dose or duration of therapy. Further, the clinical and/or biochemical features that would constitute therapeutic failure or disease relapse are also not provided, which is important when up to 42% of patients with NXG are known to relapse within 6–12 months of treatment.^1^ Guidance regarding second‐ and third‐line therapeutic options are scant, with few reports recommending intravenous immunoglobulin, infliximab and extracorporeal photopheresis as potential considerations.^11^, ^12^, ^13^

We present the case of an elderly Caucasian gentleman with extensive, upper truncal NXG lesions and an associated IgG kappa paraproteinemia who responded to combination melphalan/prednisone therapy. The therapeutic challenges of NXG are considered. The case highlights that a prolonged course of alkylating therapy combined with corticosteroid is capable of achieving disease regression in NXG. Further, clinical and biochemical correlates defining disease relapse are offered, suggesting the need to review published treatment and monitoring guidelines for NXG.

## 2 CASE PRESENTATION

An 84‐year‐old Caucasian gentleman was referred to a tertiary hematology clinic in late June 2021 for assessment of a monoclonal paraproteinemia and worsening truncal skin lesions. He reported a four‐year history of progressive erythematous, scaly lesions across his bilateral shoulders and scapulae, which had started to increase in size in the preceding three months. He complained of associated pruritus, but no pain or ulceration. He denied any fevers, drenching night sweats, unintentional weight loss, constitutional symptoms, petechiae, bone pains, bleeding diatheses, recent travel outside of Australia or exposure to pets. There was no family history of any hematological diseases. He was a non‐smoker and did not consume alcohol. His past medical history included a previous myocardial infarction secondary to a total, mid left anterior descending artery occlusion for which he had a drug eluding stent inserted, hyperlipidaemia, type 2 diabetes mellitus, atrial fibrillation complicated by a previous ischaemic stroke and splenic infarct, left carotid artery stenosis which had been stented, complex partial seizures, a small, left supramarginal gyrus multinodular and vacuolating neuronal tumor, iron deficiency requiring parenteral iron support, shingles, a provoked right upper limb deep vein thrombosis, and, a previous inguinal hernia mesh repair. His chronic medications included apixaban 2.5 mg bd, aspirin 100 mg daily, atorvastatin 40 mg daily, bisoprolol 2.5 mg daily, metformin 1 g daily, and levetiracetam 1 g mane and 1.5 g nocte. He was generally fit and well and resided at home with his wife.

**FIGURE 1:**
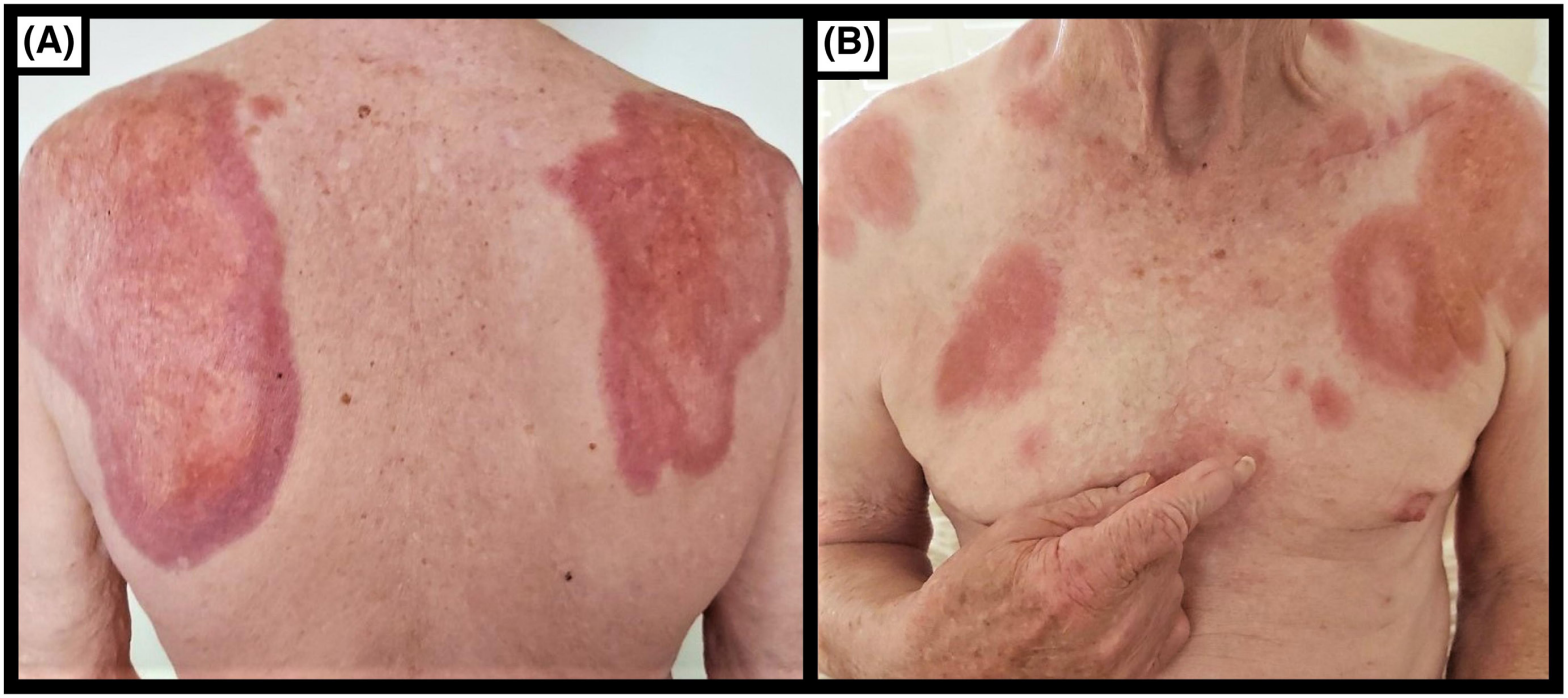
Pre‐treatment necrobiotic xanthogranuloma skin lesions across the patient's (A) upper trunk and (B) bilateral scapulae demonstrating severe erythema and induration without ulceration.

He examined as an elderly gentleman with a normal BMI. Large, confluent red‐yellow, indurated patches and plaques without ulceration were noted across his bilateral scapulae, right anterior neck, left upper chest and shoulder (Figure 1). There were no periorbital lesions seen. There was no hepatosplenomegaly or lymphadenopathy.

**FIGURE 2:**
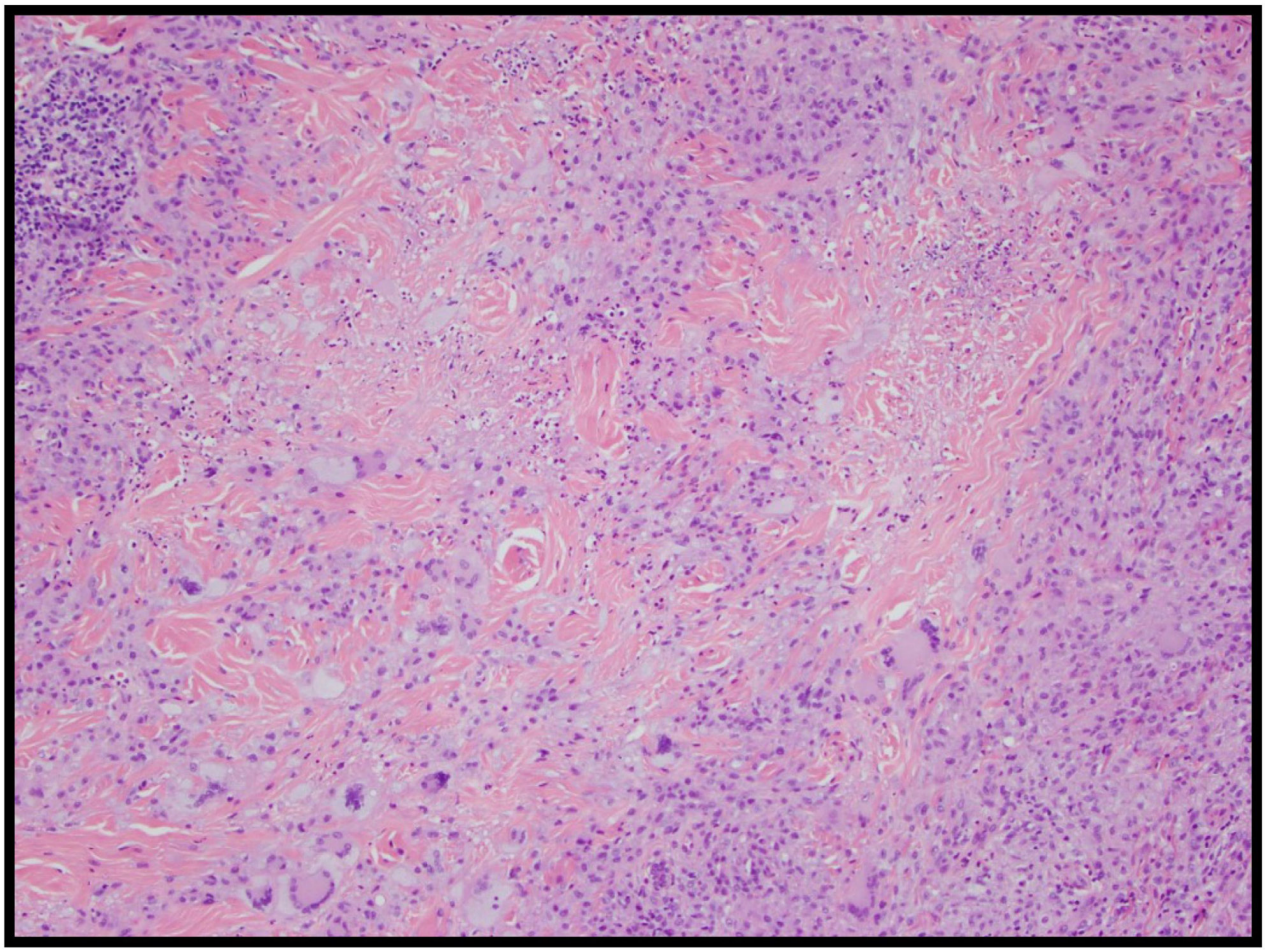
Left upper scapula skin lesion biopsy: Classic histological features of necrobiotic xanthogranuloma are shown. Necrobiotic zones with foci of leucocytoclasis, histiocytes, including foamy histiocytes, and giant cells with some Touton features are seen. Hematoxylin and eosin stain, 10× magnification.

Concurrent dermatology review was obtained and, following biopsy of these skin lesions to exclude other typical lesions (e.g., skin malignancies, infective etiologies, Erdheim‐Chester disease, atypical rheumatoid nodules), features were determined to be consistent with NXG (Figure 2). Given the significant association of this disease with hematological and solid tumor malignancies,^1^, ^2^ he underwent a comprehensive blood, bone marrow, and radiological screen. His full blood count parameters showed a mild microcytic, normochromic anemia (hemoglobin 112 g/L, MCV 79 fL, MCHC 331 g/L) with normal leukocytes (7.89 × 10^9^/L) and platelets (235 × 10^9^/L). His renal and liver functions were generally unremarkable with a normal creatinine (79 μmol/L), eGFR 78 ml/min/1.73m^2^ and only mildly elevated globulins of 43 g/L (reference range: 25–42 g/L). His calcium level was normal. His erythrocyte sedimentation rate (ESR) was elevated (66 mm/h, reference range: 1–30 mm/h), as was his C‐reactive protein (31 mg/L, reference range <5.0 mg/L) and fibrinogen (6.2 g/L, reference range: 2.0–4.0 g/L). An autoimmune screen, including a rheumatoid factor, and anti‐CCP were both negative. His lipid profile was previously unremarkable except for a mildly suppressed HDL (0.5, reference range: >1.0). His immunoglobulin G (IgG) level was raised (20.9 g/L, reference range: 5.8–13.7 g/L) with normal IgA and IgM serum levels. Subsequent serum protein electrophoresis and immunofixation identified a monoclonal IgG kappa paraproteinemia (13 g/L). His serum free kappa was quantified at 21.9 mg/L (reference range: 3.3–19.4 mg/L) with a normal free kappa/lambda ratio of 1.60 (reference range: 0.26–1.65). His beta‐2‐microglobulin was marginally elevated at 3.1 mg/L (reference range: <2.9 mg/L). Computerized tomography imaging of his chest, abdomen, and pelvis did not reveal any lymphadenopathy, hepatosplenomegaly, or visceral organ involvement of his NXG. Recent endoscopic investigations had excluded a bowel malignancy. He proceeded to a bone marrow biopsy that showed a mildly hypercellular, reactive bone marrow with normal trilineage hematopoiesis and a small (2%) monoclonal plasma cell population. These features were consistent with a diagnosis of monoclonal gammopathy of uncertain significance (MGUS) in the context of his NXG.

**FIGURE 3:**
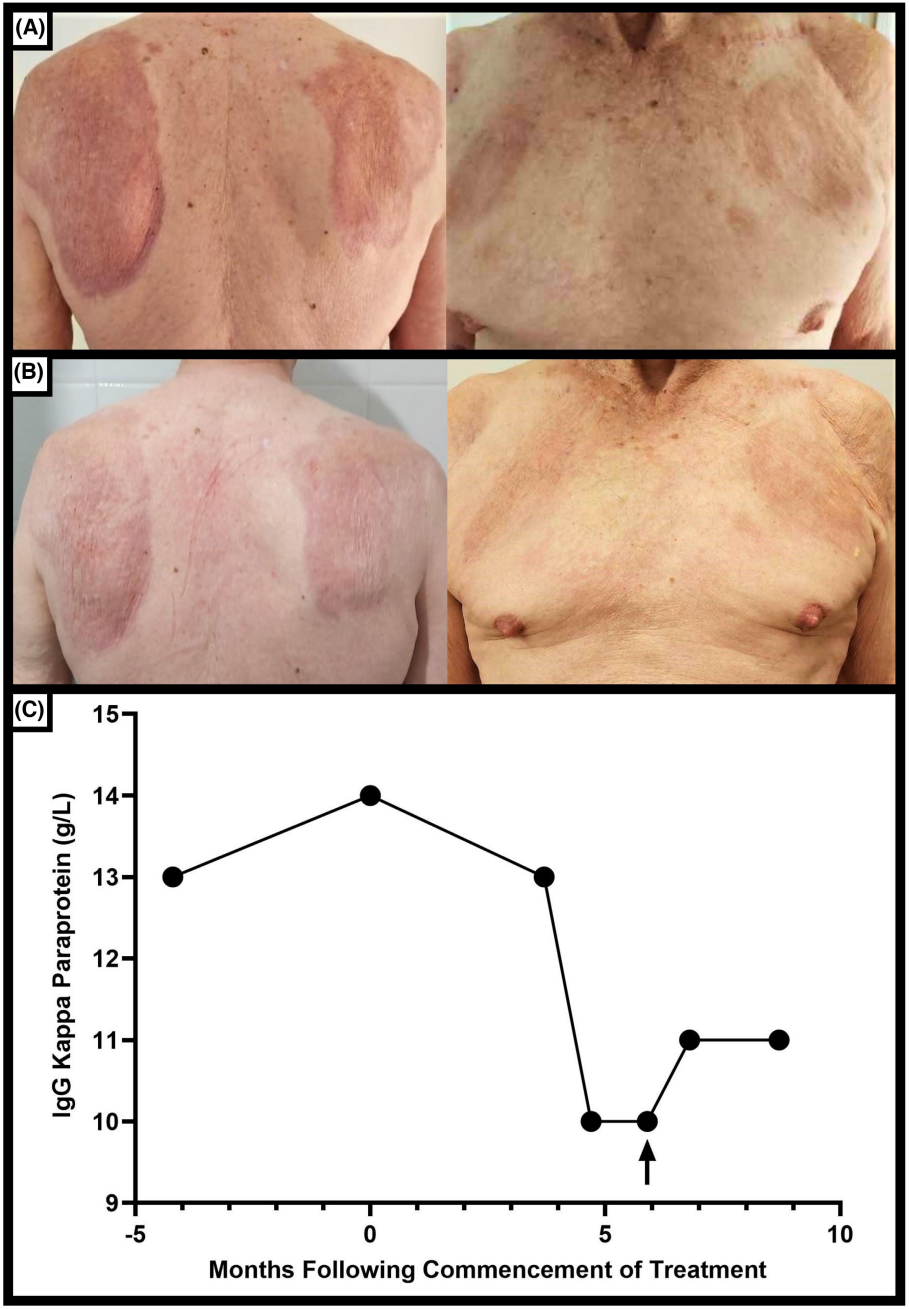
(A) Longitudinal improvement in the patient's necrobiotic xanthogranuloma skin lesions following six cycles of combination melphalan and prednisone therapy. (B) Ongoing disease regression are observed three months following treatment cessation. (C) IgG‐kappa paraprotein levels decreased and remained suppressed compared to baseline following six cycles of combination melphalan and prednisone treatment (arrow indicates completion of the sixth cycle of therapy).

During this period of investigation, his NXG lesions progressed with worsening pruritus, and he developed a new, periumbilical lesion. Given the ongoing and rapid progression of his NXG lesions across multiple skin areas, oral alkylating and corticosteroid therapy treatment was recommended. He commenced treatment three months later with melphalan 0.15 mg/kg (10 mg) daily and prednisone 25 mg daily for a total of five days out of every 28‐day cycle. He tolerated this treatment without any major adverse effects, except for some mild diarrhea with each cycle while taking melphalan. His skin lesions regressed significantly (Figure 3A,B) since his second cycle of treatment with resolution of his skin erythema, induration and pruritus. Concurrently, his paraproteinemia also reduced (Figure 3C). He developed a right upper eyelid lesion after cycle three of treatment, necessitating a skin biopsy to exclude periorbital NXG. This returned a diagnosis of a nodular basal cell carcinoma which was completed excised. He completed a total of six cycles of therapy and at three months following his last treatment, he has maintained an adequate disease response without a flare of his NXG lesions.

## 3 DISCUSSION

The clinical manifestations, treatment, and ongoing monitoring of patients with NXG are both heterogenous and complex. Treatment options are varied and depend on the coexistence of hematological neoplasms, location of dermal lesions, and consideration of visceral involvement.^1^, ^10^ In patients with extensive dermal disease and/or multisystem involvement, systemic therapies are recommended, e.g., alkylating agents, corticosteroids.^1^, ^10^ We have reported a rare case of an elderly gentleman with extensive truncal NXG and IgG‐kappa paraproteinemia that showed significant improvement following six cycles of melphalan and prednisone combination therapy. The case highlights the utility of combining alkylating agents with corticosteroids to regress NXG lesions.

Several case reports have trialed alkylating therapies with and without corticosteroids in patients with NXG.^2^, ^3^, ^4^, ^5^, ^6^, ^7^, ^8^, ^9^, ^10^ The first of these was published in 1985, in which a 66‐year‐old gentleman with NXG and IgG‐kappa paraproteinemia received melphalan 0.15 mg/kg for 5 days every 4 weeks.^3^ He received a total of three courses and achieved complete remission for 4 months, before relapsing. Others have shown similar success in using melphalan administered at similar doses in conjunction with prednisone, with some patients receiving up to six cycles of treatment.^2^, ^4^, ^5^, ^8^, ^9^ These patients showed significant regression in their NXG lesions with some attaining complete remission up to 19 months after their final treatment. We have demonstrated a similar response in our patient with six cycles of melphalan at a dose of 0.15 mg/kg daily and moderate dose prednisone, which would be most in keeping with the treatment recommendations published by Hilal et al.^10^ Only one case report has demonstrated a 65‐year‐old female with a chronic, 28‐year history of NXG that failed to respond to melphalan/prednisone therapy, with worsening of her skin lesions.^6^ It is likely that the latter is an anomaly and may be related to the extensive persistence of her disease over 28 years, whereas other case reports have detailed a shorter disease history.

The pathogenetic processes underpinning NXG are poorly understood. Two theories have been proposed; both of which are reliant on the patient having a concurrent monoclonal paraproteinemia.^2^ The first is that these paraproteins may function as “pseudo”‐lipoproteins which bind lipoprotein receptors on macrophages, stimulating granulomatous inflammation and xanthoma formation. Alternatively, these paraproteins complex with immunoglobulins externally and deposit in the skin. These complexes are recognized by the innate immune system as “foreign‐bodies,” driving a macrophage predominant response and granuloma formation.^2^ Melphalan is an attractive therapeutic option given its direct apoptotic effects on a number of cell lines, including macrophages and lymphocyte progenitors.^14^, ^15^ It, coupled with prednisone, likely inhibits granulomatous inflammation and monoclonal paraprotein production, thereby inducing disease control in patients with NXG. Optimal dosage and duration of therapy are unclear and may require individualisation.

There is a paucity of literature determining treatment failure in NXG and the clinical and/or biochemical features constituting disease relapse. Up to 42% of patients with NXG may relapse within 6–12 months of initial treatment, with relapsed NXG often demonstrating more aggressive visceral infiltration.^1^ Recurring dermal lesions with severe skin induration and ulceration should be considered as a feature of NXG relapse.^2^ Further, Wood et al.^2^ report that hematological malignancies usually arise in a large proportion of NXG patients with a monoclonal paraproteinemia within ~2.4 years. Therefore, a rising paraproteinemia requires consideration of not only NXG relapse but also the development of a de novo haematologic neoplasm. Repeat whole‐body imaging (e.g., PET‐CT scan) with supplemental lymph node and/or bone marrow biopsies should be considered. Disease relapse in patients with extensive dermal and/or visceral involvement such as ours could be potentially treated with alternative alkylating agents (e.g., chlorambucil), lenalidomide (with or without dexamethasone) or immunoglobulin.^10^, ^11^, ^12^, ^16^ The latter is an attractive option given its accepted tolerability and few side effects. Further, in patients with NXG and an associated paraproteinemia, it may function to abrogate macrophage function by reducing granulomatous inflammation, but also reduce macrophage responses to autoantibodies (e.g., paraprotein).^17^, ^18^, ^19^

Necrobiotic xanthogranuloma is a complex disease with a heterogenous presentation. In elderly patients with extensive dermal NXG and an IgG‐kappa paraproteinemia, combination melphalan/prednisone therapy provided over six cycles every 28 days is both safe and tolerable. This treatment regimen appears to induce significant disease regression and reduce monoclonal paraproteinemia in patients with NXG.^2^, ^4^, ^5^, ^8^, ^9^ Close and lifelong monitoring for disease relapse both clinically and biochemically is required, while the ongoing need to exclude new hematological malignancies, particularly with disease relapse, is of paramount importance. Future treatment guidelines are required to establish recommended, dose‐specific treatment regimens and their duration, relapsed disease monitoring requirements, and second‐line therapeutic options.

## AUTHOR CONTRIBUTIONS

JAJM and JC cared for the patient and collected longitudinal data. JAJM and JC analyzed and synthesized all the data. JAJM undertook a comprehensive literature review. JAJM wrote the manuscript. All authors critically revised and approved the final manuscript.

## CONFLICT OF INTEREST

The authors declare that they have no conflicts of interest.

## CONSENT

Written informed consent for the publication of this case and the accompanying images was obtained from the patient.

## Data Availability

All data generated and pertaining to this case report are included in the manuscript.
